# Mathematical models in GnRH research

**DOI:** 10.1111/jne.13085

**Published:** 2022-01-25

**Authors:** Margaritis Voliotis, Zoe Plain, Xiao Feng Li, Craig A. McArdle, Kevin T. O’Byrne, Krasimira Tsaneva‐Atanasova

**Affiliations:** ^1^ Department of Mathematics and Living Systems Institute College of Engineering, Mathematics and Physical Sciences University of Exeter Exeter UK; ^2^ Department of Women and Children’s Health School of Life Course Sciences King’s College London London UK; ^3^ Laboratories for Integrative Neuroscience and Endocrinology School of Clinical Sciences University of Bristol Bristol UK

**Keywords:** biophysical modelling, GnRH, mathematical modelling

## Abstract

Mathematical modelling is an indispensable tool in modern biosciences, enabling quantitative analysis and integration of biological data, transparent formulation of our understanding of complex biological systems, and efficient experimental design based on model predictions. This review article provides an overview of the impact that mathematical models had on GnRH research. Indeed, over the last 20 years mathematical modelling has been used to describe and explore the physiology of the GnRH neuron, the mechanisms underlying GnRH pulsatile secretion, and GnRH signalling to the pituitary. Importantly, these models have contributed to GnRH research via novel hypotheses and predictions regarding the bursting behaviour of the GnRH neuron, the role of kisspeptin neurons in the emergence of pulsatile GnRH dynamics, and the decoding of GnRH signals by biochemical signalling networks. We envisage that with the advent of novel experimental technologies, mathematical modelling will have an even greater role to play in our endeavour to understand the complex spatiotemporal dynamics underlying the reproductive neuroendocrine system.

## Graphical abstract

This review article provides an overview of mathematical modelling in GnRH research. We review mathematical models that have been used to describe and explore various aspects of the reproductive neuroendocrine system, including the the electrophysiology of the GnRH neuron; GnRH signalling in the pituitary; and the mechanisms underlying GnRH pulsatile secretion.

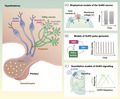

## 1 INTRODUCTION

The reproductive axis is a complex regulatory system: it spans multiple levels of organisation (from molecular and cellular to organ and organismal levels);^1^ feedforward and feedback interactions run across these levels at multiple timescales (ranging from minutes to days);^2^and there is complex crosstalk with other endocrine axes and the central nervous system.^3^, ^4^ These factors hamper our intuition regarding the system especially when it comes to the system's dynamical behaviour in normal physiological conditions or under acute perturbations and chronic disease. In face of these challenges, mathematical modelling is an indispensable tool for solidifying our understanding of the system, gaining insight into its behaviour, and designing how to tackle specific research questions.

**FIGURE 1:**
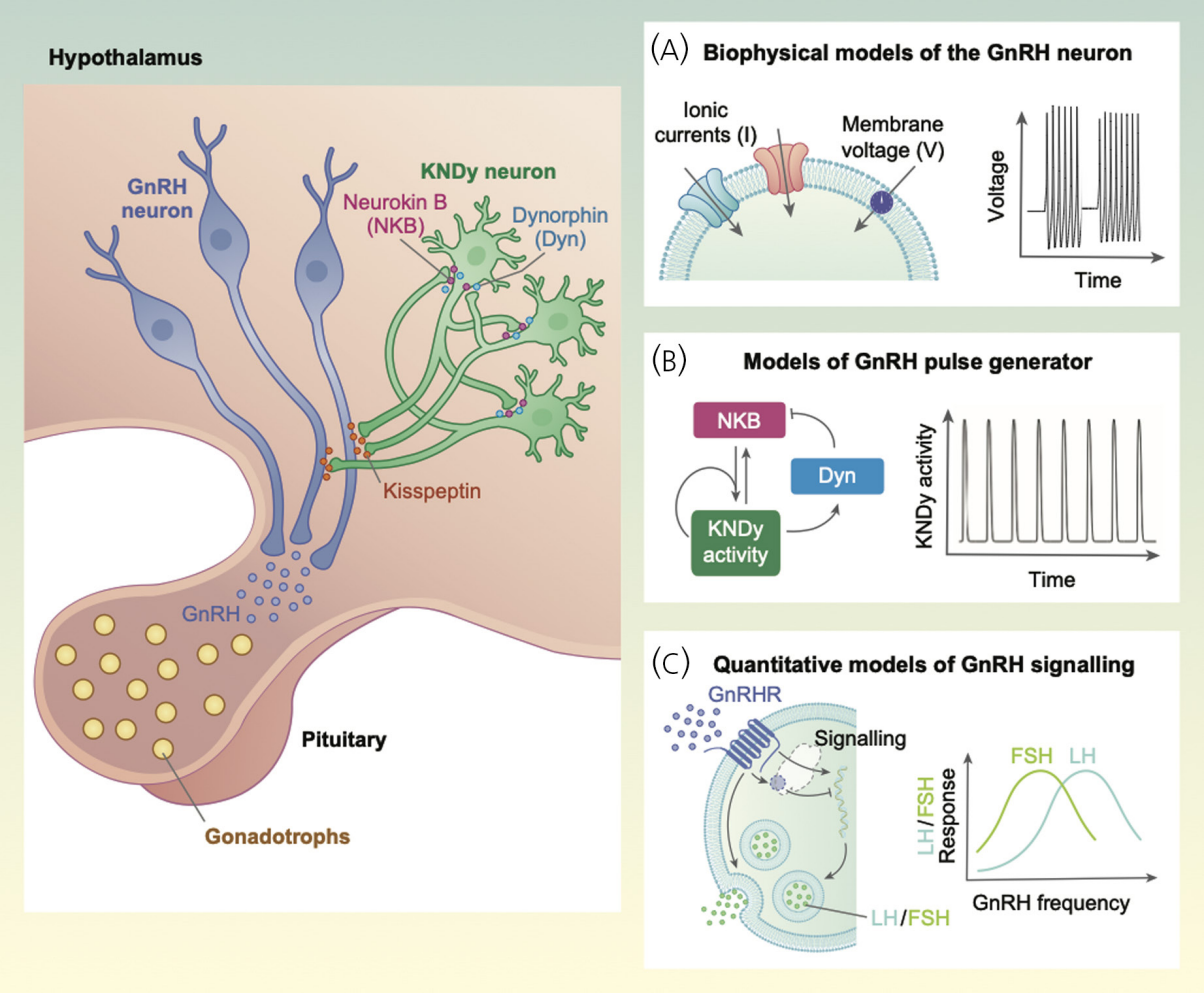
Overview of mathematical models in GnRH research. Mathematical modelling has been used to describe and explore various aspects of the reproductive neuroendocrine system including (A) the electrophysiology of the GnRH neuron and its bursting in vitro activity, (B) the mechanisms underlying GnRH pulsatile secretion, and (C) GnRH signalling to the pituitary and the nonlinear effects of GnRH frequency on gonadotropin secretion

The aim of this article is to provide an overview of the mathematical modelling work in the context of GnRH research (Figure 1). Rather than try to cover the entire literature of mathematical models in the field, we chose to present how mathematical models have coevolved with our understanding of the reproductive neuroendocrine system following the discovery of GnRH,^5^ and how models have contributed to our current understanding of the system. Hence, this review focuses on the analysis and interpretation of mathematical models and not on technical details underpinning their development and analysis. For more technical yet accessible reviews of mathematical modelling in neuroendocrinology we refer the reader to two previous studies.^6^, ^7^ The review is based on three key research areas where mathematical modelling has been particularly relevant: the GnRH neuron, GnRH signalling to the pituitary, and GnRH pulsatile secretion.

## 2 A QUANTITATIVE DESCRIPTION OF THE GNRH NEURON

In vitro studies using GnRH model systems and acute brain slice preparation have suggested that sustained bursting activity and spontaneous Ca^2+^ transients are key aspects of the GnRH neuron behaviour.^8^, ^9^, ^10^, ^11^, ^12^ To understand the mechanisms underlying bursting activity several biophysical models have been proposed in the literature.^9^, ^13^, ^14^, ^15^ Using the formalism developed by Hodgkin and Huxley in their seminal work of characterising the squid giant axon,^16^ these models describe how inward and outward currents, conducted through voltage‐gated ion channels, contribute to membrane voltage changes and action potential generation. As these models were constructed based on data from different biological models of the GnRH neuron, a consensus on the importance of different ion channels and other system parameters is difficult. However, all of the models suggest that intracellular Ca^2+^ dynamics and slow Ca^2+^ currents are crucial for bursting activity in GnRH neurons, a finding that was experimentally verified using dual electrical‐Ca2+ recordings from acute mouse brain slices.^10^

Moreover, a mathematical model has been proposed to understand the origin of the two distinct types of bursting: parabolic bursting, where the spike frequency profile during the active phase resembles a downward‐opening parabola, and irregular bursting, where there is marked variability in the observed interburst interval and burst duration.^15^ A key innovation of this model was the inclusion of biological noise leading to spontaneous action potentials. The model accurately reproduced both parabolic and irregular bursting (in terms of active phase duration, spike count, and interspike interval and interburst interval) as the ion channel conductance parameters were changed, suggesting that the mode of bursting should depend on the relative ion channel expression on the cell membrane. This theoretical finding emphasises a key pathway through which neuromodulators, such as gonadal steroids and kisspeptin, could change the electrical behaviour of the GnRH neuron.^17^

A unique morphological characteristic of GnRH neurons is their long dendritic‐like projections that lead into the median eminence where they break up into short axonal terminals.^18^ These projections function both as dendrites and axons, hence dubbed “dendrons”, as they have the capacity to receive synaptic inputs as well as conduct action potentials to facilitate GnRH secretion from their terminals.^18^ A stochastic spatiotemporal model of the dendron has been proposed to study the functional relevance of this structure.^19^ The model predicts that stochastic synaptic inputs along the dendron could be crucial for action potential initiation but not action potential propagation to the terminals. The morphology of the GnRH neuron also affects the architecture of the GnRH neuronal network with dendrons bundling up as they converge to the median eminence.^20^ These unique features would require novel mathematical models, extending the Hodgkin‐Huxley formalism, to fully capture their functioning and shed light on their biological relevance.

## 3 MODELLING GnRH SIGNALLING

Hypothalamic GnRH signals are decoded in the anterior pituitary gland by gonadotropes, leading to the synthesis and secretion of gonadotropin hormones: luteinizing hormone (LH) and follicle‐stimulating hormone (FSH).^21^ Early experimental work by the Knobil laboratory revealed the importance of GnRH frequency for gonadotropin secretion, showing that in primates with hypothalamic lesions it is possible to restore gonadotropin secretion with pulsatile (in contrast to constant) delivery of exogenous GnRH.^22^ Although it is now clear that gonadotropin synthesis and secretion are suppressed when GnRH frequency is either too high or too low, there are still open questions regarding what mechanisms underly this effect.^21^ A key feature of the GnRH decoding process is that cellular responses are often maximal for intermediate GnRH pulse frequencies^21^, ^23^ and in vitro work with pituitary cell cultures (LβT2 cells) has shown that the expression of different genes involved in GnRH signalling and LH/FSH secretion (LHβ, FSHβ, αGSU, GnRHR) is maximized at different GnRH pulse frequencies.^24^ Importantly, differences in these frequency‐response relationships could be of relevance in fertility related conditions, as for example in women with polycystic ovarian syndrome, where the increased frequency of GnRH pulses is thought to increase secretion of LH while suppressing FSH secretion and disrupting the reproductive cycle.^25^ A mathematical model has proposed that such differences between LH and FSH secretion patterns in response to GnRH frequency could be explained through the signalling action of polypeptides activin and follistatin.^26^ Furthermore, mathematical modelling suggests that bell‐shaped pulse frequency‐response relationships reflect a signalling architecture that incorporates either negative feedback loops or feedforward control motifs (where a target is differentially regulated through distinct branches).^27^

To elucidate the mechanisms underlying GnRH effects, a model incorporating negative feedback in the form of agonist‐induced GnRH receptor internalization was developed and simulated, showing that high internalization rates could result in nonmonotonic relationships.^28^ However, the pronounced levels of desensitization predicted by the model are in disparity with wet‐laboratory data, and hence the model argues against internalisation being a credible explanation of the bell‐shaped pulse frequency‐response relationship. Other negative feedback mechanisms that could be responsible for the nonmonotonic GnRH effects include: GnRH‐stimulated RGS protein expression,^29^ MKP expression,^30^ IP_3_ receptor downregulation^31^, ^32^ as well as ERK‐mediated negative feedback.^33^, ^34^ Furthermore, a theoretical study showed that receptor dimerization upon GnRH binding (effectively a form of negative feedback via receptor sequestration) could also generate nonmonotonic relationships.^35^ However, it still remains unclear if these mechanisms are relevant for GnRH frequency decoding.

Feedforward motifs are ubiquitous structural elements in signalling and gene expression networks.^36^ In feedforward motifs, two (or more) regulatory branches fan out from an upstream node and converge at a downstream node. If these two branches have the same regulation sign (i.e., both stimulatory or both inhibitory) the structure is termed coherent feedforward motif (CFFM), and when the two branches have different signs (i.e., one is stimulatory and the other is inhibitory), it is termed incoherent feedforward motif (IFFM). Theoretical investigation has showed that for a broad range of pulse frequencies, CFFMs generate monotonic frequency‐response relationships, whereas IFFMs yielded nonmonotonic frequency‐response relationships.^37^ Therefore, IFFMs provide a credible mechanism underlying the bell‐shaped frequency‐response relationship observed in GnRH decoding by gonadotropes. Examples of feedforward motifs within the GnRH signalling network is the activation of conventional PKC isoforms by PLC‐mediated Ca^2+^ mobilization and DAG production,^38^, ^39^, ^40^, ^41^, ^42^ but CFFMs and IFFMs are also relevant in gene expression where multiple GnRH‐regulated transcription factors regulate the expression of genes.

## 4 INFORMATION TRANSFER IN GNRH SIGNALLING TO GONADOTROPES

Heterogeneity is ubiquitous in biological systems, and even genetically identical single cells can exhibit striking variation in protein expression levels and in their sensitivity to external stimulation.^43^ Heterogeneity has been explored in the context of gonadotropes and has shown that GnRH can have a variable effect on many cellular responses, such as gonadotropin secretion, ERK activation, calcium dynamics, and gene expression.^33^, ^44^, ^45^, ^46^, ^47^, ^48^, ^49^, ^50^, ^51^ Information theory provides a quantitative toolset to study how this variability impacts on the ability of gonadotropes to process and decode GnRH signals.^52^, ^53^

**FIGURE 2:**
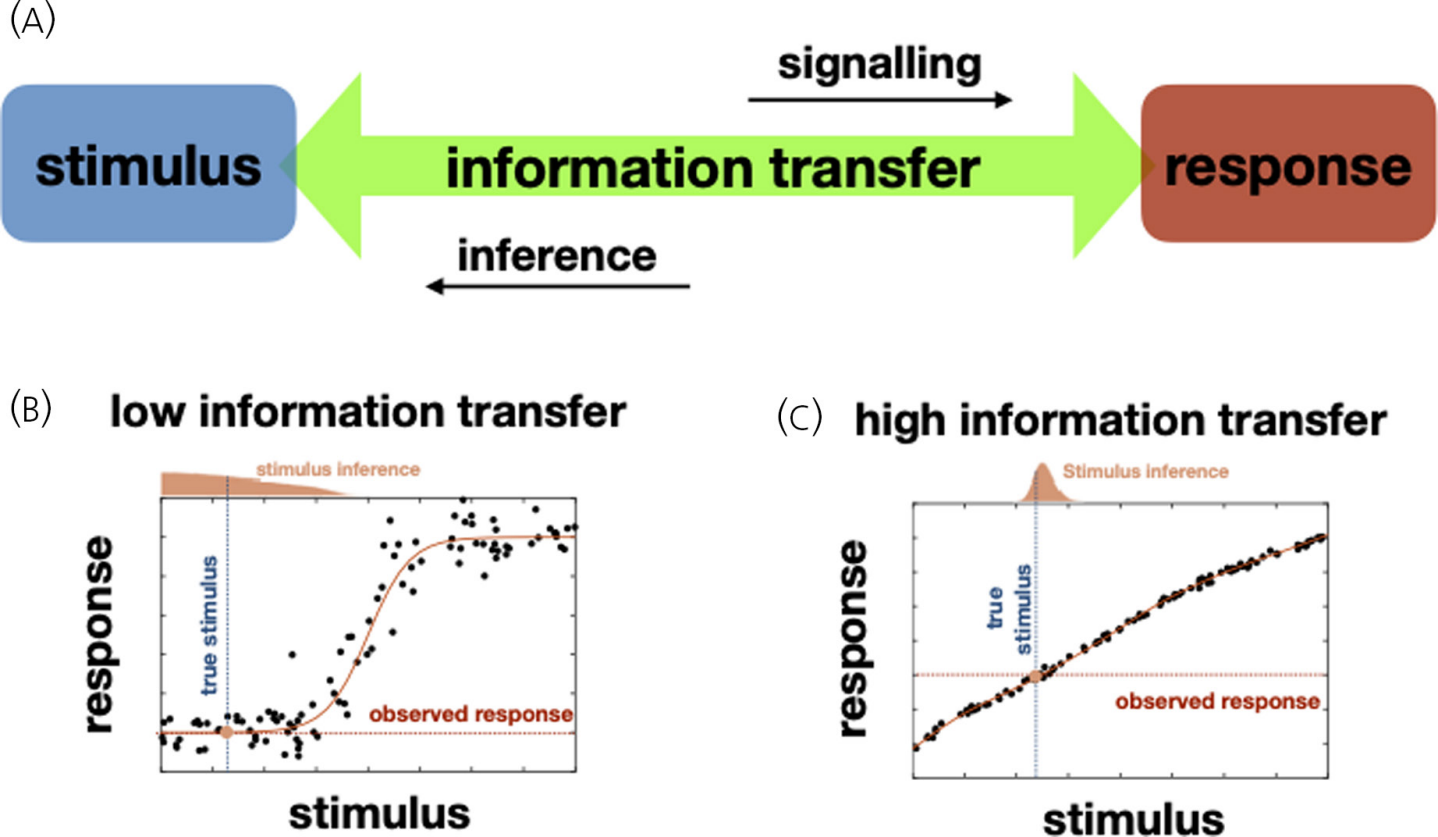
Information transfer in cell signalling systems. (A) Cellular responses are variable and hence signalling pathways can be conceptualised as noisy communication channels. Information transfer measures how reliably an environmental stimulus can be inferred from the observed cellular response. (B) For a signalling system with low information transfer, cellular responses measured at different stimulation levels (measurements represented by black dots) show high variability across cells and differ significantly from the average (represented by the solid red line). Identifying the true stimulus value (vertical dotted line) for an observed response (horizontal dotted line) is difficult as the uncertainty of the inference is large (wide inferred distribution). (C) For a signalling system with high information transfer, responses are less variable and the true stimulus level (vertical dotted line) can be inferred with greater accuracy (narrow distribution) from the observed response (horizontal dotted line). For these illustrations inference is performed using the Bayesian framework resulting in a posterior distribution for the stimulus. A uniform prior distribution for the stimulus is assumed

In any communication system, where a message is transferred to a receiver, information transfer can be defined as the reduction in uncertainty regarding the contents of the message that is accomplished through the communication channel. In particular, the information transfer though a channel can be quantified using the statistical notion of mutual information (MI), which measures (usually in units of Bits) the uncertainty reduction that communication confers. Putting these statistical ideas to work in the context of cellular signalling, the message corresponds to the extracellular environment, the channel to the signalling pathway mediating the response, and the amount of information transferred measures how reliably the environment can be inferred from the cellular responses (Figure 2).

Cell‐to‐cell variability and the inherent stochasticity underlying biochemical reactions renders signalling pathways noisy communication channels and therefore prone to information loss. This has been extensively studied in vitro in the context of GnRH signalling. ERK phosphorylation and Egr‐1 driven gene expression were used as two distinct readouts of GnRH signalling and measured in millions of individual cell transfected with GnRH receptors (GnRHR) under 8 GnRH concentrations.^51^ The maximum information transfer in this setup is 3 Bits (being able to discriminate all 2^3^ = 8 GnRH concentrations); however, the measured MI values indicated that information transfer achieved through GnRHR is less than 1 Bit, implying that single cells cannot unambiguously distinguish between two conditions (i.e., with and without GnRH) similar to what is observed for cytokine and growth factor signalling.^54^ Low values of MI (typically <0.5 Bits) were also obtained using NFAT‐EFP and NFAT response‐element driven fluorophore expression as readouts for activation of the Ca^2+^ to NFAT pathway, both in GnRHR transfected cells and in LβT2 cells with endogenous GnRHR.^51^, ^55^ Finally, MI values were increased by consideration of joint signalling (i.e., using readouts from both ERK and NFAT pathways) but still did not exceed 1 Bit.^51^

In vitro studies have also started to explore information transfer by sensing GnRH dynamics.^52^, ^53^ To accomplish this, GnRH‐stimulated nuclear translocation of NFAT‐EFP was monitored in live cells following short (5 min) GnRH pulses.^55^ Using the entire response trajectories after a GnRH pulse to calculate MI yielded values that were marginally higher than those calculated using snap‐shot data (i.e., responses at a single time point after the pulse) suggesting that little information is actually gained by sensing the entire response trajectory.^55^ Moreover, although responses to GnRH stimulation are markedly heterogeneous between cells, there is reproducibility when it comes to how individual cells respond to successive GnRH stimulations over the experimental time frame (2 h). By means of a stochastic model of GnRH signalling to NFAT, this experimental observation suggests that the signalling machinery (e.g., concentrations of the GnRHR and calmodulin) remains relatively stable in individual cells but varies within the population.^55^ Importantly, this property can enable a cellular population to respond in a more graded fashion to GnRH stimulation despite individual cells having a limited information transfer capacity.

An interesting theoretical observation is that negative feedback has the potential to mitigate information loss in signalling cascades,^56^ and this effect has also been explored in the context of GnRH signalling.^51^ In particular, a stochastic model for GnRH‐mediated ERK activation was used to study the effect of negative feedback loops on information transfer and was shown that maximal information transfer is achieved for intermediate feedback strengths. That is, both low and high levels of feedback hamper information transfer, by allowing noise due to basal (constitutive) signalling activity in the former case; and reducing response amplitude in the latter case. This prediction was tested with a series of in vitro single cell experiments, where it was shown that information transfer could be reduced either by inhibiting ERK‐mediated negative feedback (expressing catalytically inactive ERK2) or via increasing transcription‐dependent negative feedback strength (increasing ERK‐driven MKP expression).^51^ These findings highlight the crucial role that well‐balanced negative feedback loops play in GnRH‐mediated information transfer.

## 5 THE EMERGENCE OF GNRH PULSES

Uncovering and understanding the mechanisms that drive and regulate pulsatile GnRH secretion is a key steppingstone towards a comprehensive picture of the reproductive axis and mathematical models have provided great insight into this endeavour. Pulsatile GnRH release into the anterior pituitary gland is a critical step in homeostatic control of the HPG axis, stimulating the release of gonadotropin hormones which will, in turn, drive gonadal processes and sex‐steroid regulation. An early phenomenological, data‐driven model of GnRH secretion dynamics suggested that pulse generation could emerge due to timescale differences in nonlinear feedback interactions between system components.^57^

Following the discovery of neuropeptide kisspeptin and the repressing effect the kisspeptin signalling disruption has on reproduction,^58^, ^59^, ^60^ it was immediately appreciated that hypothalamic kisspeptin releasing neurons might be key for the pulsatile secretion of GnRH. A major population of kisspeptin neurons resides in the arcuate nucleus, also known as KNDy for expressing kisspeptin, neurokinin B and dynorphin, and it has been shown to be responsible for generating pulses of activity driving LH secretion in mice.^61^ Complementing this experiment evidence, a mathematical model of the KNDy population provided insight into the mechanisms enabling pulsatile dynamics.^62^ The model incorporated neuropeptide‐driven interaction between KNDy neurons, namely the excitatory effect of neurokinin B on neuronal dynamics and the inhibitory effect of dynorphin.^63^ Analysis of the model showed that KNDy neurons drive GnRH pulses by operating collectively as a relaxation oscillator, due to the positive and negative feedback interactions that are generated through neurokinin B and dynorphin signalling, respectively. Furthermore, the model suggested that pulsatile dynamics should critically depend on the levels of basal activity in the kisspeptin population. In particular, the model predicted that as basal activity is progressively increased, the system's dynamics undergo a qualitative change from a quiescent into a pulsatile state. To confirm this model prediction, optogenetics were used to selectively activate KNDy neurons at different frequencies (0.5–20 Hz), emulating in this manner an increase in their basal activity. The results showed that sustained low‐frequency (1–5 Hz range) optic stimulation is sufficient to trigger robust LH pulses in oestrous mice.^62^ This finding illustrates how GnRH pulses can be regulated via changes in the intrinsic activity of KNDy neurons (mediated by gonadal steroids) or due to persistent signals to the KNDy population from afferent neuronal populations.

The mathematical model of the KNDy population, has also been used, more recently, to infer cycle changes in four key parameters controlling pulsatile dynamics: (i) dynorphin signalling strength, (ii) neurokinin B signalling strength, (iii) level of network excitability and (iv) basal neuronal activity. The model was trained on experimental observations of LH frequency (as a proxy of GnRH frequency) from oestrous and dioestrous mouse with and without low‐frequency optic stimulation at 5 Hz. The model predicted that network excitability controlled via glutamatergic transmission is a key regulator that covaries along with neurokinin B and dynorphin across the oestrous cycle.

Despite KNDy being critical for the generation of GnRH pulses, the possibility that GnRH frequency is also modulated by factors downstream of KNDy cannot be excluded, and this scenario was investigated by a recent mathematical model of the GnRH neuron.^64^ The model builds upon previous modelling attempts based on the observation that immortalized GnRH‐secreting neurons (GT1‐7 cells) express GnRH receptors enabling GnRH to control intracellular levels of Ca^2+^ and cAMP and hence potentially autregulate their secretion rate.^65^, ^66^, ^67^ Analysis of the model predicted that this autocrine signalling could potentially filter out kisspeptin pulses, making the frequency of GnRH release (approximately 7‐fold) slower than the frequency of the kisspeptin input. However, it should be noted that the one‐to‐one correspondence between KNDy activation events (proxy for kisspepetin release) and LH pulses (proxy for GnRH release) observed in intact female and gonadectomised male mice,^61^, ^68^ suggests that in these contexts the GnRH autocrine feedback is either absent or its timescale is fast relative to the endogenous kisspeptin pulses.^7^ Nonetheless, the model raises interesting questions as to whether nonlinear mechanisms operating at the level of the GnRH neuron (rather than the gonadotrope) could also be contributing to the nonlinear relationship observed between pulse generator and pulsatile LH output when the pulse generator frequency is^69^ high.^23^, ^69^

## 6 CONCLUSION AND PROSPECT

Mathematical models have proven remarkably helpful in our endeavours to dissect the complexity of the reproductive neuroendocrine system and understand its dynamic behaviour. The development of these mathematical models has been intricately linked to advancements in experimental methods and technologies, enabling a deeper understanding of the GnRH system. In vitro electrophysiological studies have provided crucial information for the development of detailed biophysical models of the GnRH neuron leading to a quantitative understanding of the bursting patterns of electrochemical activity observed. Moreover, live cell microscopy has enabled the development of mechanistic models of GnRH signalling allowing us to understand properties of the biochemical pathways enabling reliable transmission of GnRH information to the pituitary. Recently, advances in optogenetics has sparked the development of mathematical models describing the dynamic behaviour of neuronal populations involved in pulsatile GnRH release.

As technology will continue to evolve so will our capacity to formulate more accurate models providing further insight into the reproductive neuroendocrine system. Towards this direction, transcriptome profiling studies of hypothalamic neurons^70^, ^71^ will be crucial in refining our current quantitative models of the system and uncovering the mechanisms allowing GnRH regulation by gonadal steroids. Furthermore, the advent of in vivo methods monitoring neuronal activity at the single neuron level (microendoscopy) will require more detailed neuronal population models that will enable us to understand how cells communicate and synchronise their activity to achieve pulsatile GnRH release.^69^ Likewise, with the advent of real‐time hormonal monitoring technologies^72^ the development of parsimonious yet reliable models will become even more relevant for the clinical care of patient with reproductive disorders, handling real‐time data analysis and feeding back to the care protocol.

## CONFLICT OF INTEREST

The authors declare that there is no conflict of interest.

## AUTHOR CONTRIBUTIONS

**Margaritis Voliotis:** Visualization; Writing – original draft. **Zoe Plain:** Resources; Software; Visualization. **Xiao Feng Li:** Writing – review and editing. **Craig A. McArdle:** Writing – review and editing. **Kevin Thomas O'Byrne:** Writing – review and editing. **Krasimira Tsaneva:** Writing – review and editing.

### PEER REVIEW

The peer review history for this article is available at https://publons.com/publon/10.1111/jne.13085.

## Data Availability

No unpublished data have been presented.
